# Associations of sleep disturbances in systemic lupus erythematosus with physical and psychological outcomes: a cross-sectional latent profile analysis

**DOI:** 10.3389/fimmu.2025.1626597

**Published:** 2025-10-15

**Authors:** Ling Ma, Yan-Hong Li, Xin Guo, Ying Wang

**Affiliations:** Department of Rheumatology and Immunology, West China Hospital, Sichuan University, Chengdu, China

**Keywords:** systemic lupus erythematosus, cluster analysis, latent profile analysis, sleep quality, influencing factor

## Abstract

**Purpose:**

Patients with systemic lupus erythematosus (SLE) frequently experience poor sleep quality. This cross-sectional study aimed to identify distinct sleep disturbance profiles in SLE patients and examine their associations with demographic, disease-related, and psychosocial factors.

**Methods:**

A total of 331 patients with SLE were included. Latent profile analysis (LPA) was conducted using the tidyLPA package. Logistic regression models were constructed to assess associations between the identified sleep disturbance clusters and physical and psychological outcomes, based on factors significantly influencing the LPA results. The physical and psychological outcomes were estimated using the Hospital Anxiety and Depression Scale (HADS) and the Fatigue Severity Scale (FSS). Sleep clusters were analyzed through multivariate logistic regression.

**Results:**

Three distinct sleep disturbance profiles were identified: Cluster 1 (severe sleep disturbance) (n = 42), Cluster 2 (moderate sleep disturbance) (n = 174), and Cluster 3 (mild sleep disturbance) (n = 115). LPA yielded an entropy value of 0.996 for the three-cluster model. The mean total Pittsburgh Sleep Quality Index (PSQI) score for the SLE samples was 7.59 ± 3.44. Among the various sleep quality domains, sleep latency and subjective sleep quality were the most significantly affected in SLE patients. The analysis revealed that disease duration, severity of fatigue, use of calcium supplements, impaired renal function, anxiety, and depression were all significant factors influencing cluster membership.

**Conclusion:**

This study identified three distinct patterns of sleep disturbance among SLE patients. Cluster 1 (severe sleep disturbance) was characterized by prolonged sleep latency despite high sleep efficiency and subjective sleep quality scores. Cluster 2 (moderate sleep disturbance) exhibited longer sleep duration than Cluster 1, while Cluster 3 (mild sleep disturbance) had the lowest scores across all sleep quality domains. These findings suggest that sleep disturbance profiling may facilitate personalized sleep management strategies for patients with SLE.

## 1 Introduction

Systemic lupus erythematosus (SLE) is an autoimmune-mediated connective tissue disease characterized by immune-driven inflammation (1). Previous studies have reported that the prevalence of SLE in China ranges from 30 to 70 cases per 100,000 individuals (2). Patients with SLE generally experience a lower quality of life compared to the general population (3, 4).

Despite significant advancements in pharmacological treatments over recent decades, the health-related quality of life of SLE patients remains suboptimal (5). The current approach to SLE management has shifted toward long-term chronic disease care, emphasizing quality-of-life improvement (4), a key recommendation in the management of rheumatic diseases (6, 7).

Cluster analysis is an unsupervised data analysis technique that groups similar data points into clusters based on shared characteristics. This method helps uncover underlying structures and patterns, facilitating classification for further analysis. Compared to traditional discrete clustering methods, latent profile analysis (LPA) offers greater statistical power, improving cluster accuracy while reducing the false-positive rate. LPA employs multivariate algorithms to assess similarities between samples based on continuous indicators (8, 9), enabling their classification into distinct clusters.

Applying cluster analysis to SLE patients can help categorize individuals based on sleep patterns, allowing for the identification of distinct sleep disturbance profiles. These profiles may enhance the understanding of specific patterns and contributing factors in SLE-related sleep disturbances while also revealing similarities and differences among patients. With the increasing reliance on data-driven approaches across research fields, cluster analysis has become essential for developing personalized treatment strategies and predicting disease progression and outcomes.

This study aimed to investigate the heterogeneity of sleep disturbance profiles among SLE patients. We hypothesized that identifying common sleep disturbances within patient subgroups could provide clinicians with valuable insights for optimizing the management and treatment of sleep problems in SLE. To test this hypothesis, we conducted a cluster analysis of outpatient SLE patients to identify distinct sleep disturbance profiles, examine their associated clinical features, and explore the potential mechanisms linking sleep disorders with SLE, such as immune dysfunction or the side effects of drugs.

However, the clinical presentation of sleep problems varies widely, suggesting the existence of distinct subtypes with different therapeutic needs. Most existing studies, however, focus on a single dimension (such as insomnia severity) and lack an integrated classification based on multidimensional sleep characteristics. This raises two questions: Can subgroups with different clinical characteristics be identified by PSQI subscales, and do these subgroups require differentiated intervention strategies?

## 2 Methods

### 2.1 Study participants

This was a cross-sectional observational study that included 331 SLE patients recruited from a tertiary hospital’s rheumatology and immunology department between 2021 and 2022. Sleep and self-reported outcomes in were assessed. In previous studies we have used baseline data from this program to construct a nomogram for poor sleep quality in patients with systemic lupus erythematosus (SLE) (10). However, the current submission aims to identify distinct sleep disturbance profiles in SLE patients and examine their associations with demographic, disease-related, and psychosocial factors.

Participants were enrolled based on predefined inclusion and exclusion criteria. The inclusion criteria were as follows: (a) patients with a history of ANA positivity who met the 2019 EULAR/ACR, 2012 Systemic Lupus International Collaborating Clinics (SLICC), or 1997 ACR criteria for SLE classification (11–13); (b) age between 18 and 70 years; and (c) ability to understand the study procedures and provide written informed consent.

Patients were excluded if they met any of the following criteria: (a) presence of other autoimmune rheumatic diseases (e.g., rheumatoid arthritis, Sjögren’s syndrome, ankylosing spondylitis, scleroderma, dermatomyositis, or fibromyalgia); (b) concomitant vital organ failure or malignancy; (c) severe cognitive impairment, dementia, psychiatric disorders, or other neurodegenerative diseases; (d) suspected or confirmed pregnancy; or (e) inability to complete the study questionnaires independently or with assistance.

This study was conducted in accordance with the Declaration of Helsinki and was reviewed and approved by the Ethics Committee of West China Hospital, Sichuan University. All eligible participants were informed about the study’s purpose, procedures, potential risks and benefits, and their right to withdraw at any time.

### 2.2 Data collection

1. General Clinical Data: Collected data included demographic information, personal history (smoking and alcohol consumption), medical history (hypertension, diabetes, dyslipidemia), chief complaints, medication history, and SLE-related clinical characteristics (disease duration, initial symptoms, clinical manifestations, and organ system involvement).
2. Laboratory Data: Parameters included complete blood count, liver and kidney function tests, electrolyte levels, immunoglobulins, complement components, autoantibodies, 24-hour urine protein quantification, and urinalysis.
3. Clinical Characteristics of SLE Patients: Symptoms recorded included facial erythema and rash, headache, nausea, vomiting, joint pain, diarrhea, fatigue, Raynaud’s phenomenon, vasculitis, cough, chest discomfort and pain, fever, and lower extremity edema. Clinical characteristics were obtained from medical records and patient-reported chief complaints during clinical visits. Laboratory data were extracted from medical records. Metabolic syndrome, overweight status, low high-density lipoprotein cholesterol, and high triglycerides were defined according to the International Diabetes Federation criteria (14). Glucocorticoid use was categorized as low dose (≤7.5 mg/day) or medium-to-high dose (>7.5 mg/day) (15).
4. Assessment Scales: The study utilized the Pittsburgh Sleep Quality Index (PSQI), Hospital Anxiety and Depression Scale (HADS), Fatigue Scale, and Systemic Lupus Erythematosus Disease Activity Index 2000 (SLEDAI-2000).

SLE Activity: Disease activity was assessed using the Systemic Lupus Erythematosus Disease Activity Index 2000 (SLEDAI-2000), which includes 24 descriptors across nine organ systems. The recall period for disease activity assessment was the previous 10 days. Scores range from 0 to 105, with higher scores indicating greater disease activity (16).

Anxiety and Depression: Anxiety and depression were evaluated using the HADS, which consists of 14 items equally divided into two subscales: HADS-Anxiety (HADS-A) and HADS-Depression (HADS-D) (17). HADS is widely used to assess anxiety and depression in physically ill patients, as it excludes symptoms such as insomnia, loss of appetite, and fatigue that may be attributed to physical illness. A cutoff score of ≥8 is recommended to identify potential cases of anxiety and depression (18–21).

Fatigue Severity: Fatigue severity was measured using the Fatigue Severity Scale (FSS), which comprises nine items. The fatigue severity score is calculated as the mean of all items, ranging from 1 (no fatigue) to 7 (maximum fatigue). Alternatively, a total score of ≥36 was used as the cutoff for significant fatigue (22). The FSS is commonly used to assess fatigue in SLE patients (23).

Sleep Quality: Sleep quality was assessed using the PSQI, a 19-item questionnaire comprising seven components: subjective sleep quality, sleep latency, sleep duration, sleep efficiency, sleep disturbances, use of sleep medication, and daytime dysfunction over the past month. Each component is scored from 0 (no difficulty) to 3 (severe difficulty), with a total score ranging from 0 to 21. Higher scores indicate poorer sleep quality. Since its development, PSQI has been widely used in both clinical and non-clinical populations (24). In this study, a PSQI score ≥7 was considered indicative of poor sleep quality, while a score <7 was classified as acceptable sleep quality (25).

### 2.3 Statistical analysis

A database was established using Excel. Data entry was performed independently by two researchers, and 12 questionnaires with missing values were excluded. The final analysis was conducted on 331 valid questionnaires.

For descriptive analysis, the Shapiro-Wilk normality test was used to assess data distribution. Normally distributed variables were presented as mean ± standard deviation, while skewed data were reported as medians with interquartile ranges (25th and 75th percentiles). Categorical variables were expressed as n (%).

LPA was performed using the tidyLPA package. Model fit was evaluated using the Akaike information criterion, Bayesian information criterion, adjusted Bayesian information criterion, and entropy. The bootstrap likelihood ratio test and likelihood ratio test were applied for model comparison.

The following components of the PSQI were included in the cluster analysis: subjective sleep quality, sleep latency, sleep duration, sleep efficiency, and sleep disturbances. The use of sleep medication and daytime dysfunction were excluded due to their susceptibility to the effects of fatigue and mood disorders. Including unnecessary variables may compromise model identifiability and lead to overparameterization (26).

Variables with significant differences between clusters (p < 0.05) were incorporated into a logistic regression model to predict cluster membership probability. A two-tailed p < 0.05 was considered statistically significant.

### 2.4 Size calculation

There is no universally accepted guideline for the minimum sample size required in cluster analysis (27). However, some studies have suggested a minimum sample size of 2k, where k represents the number of variables (28). Based on this recommendation, a minimum sample size of 128 (k = 7, 27 = 128) was considered necessary to ensure the reliability and interpretability of the results in this study.

## 3 Results

### 3.1 Demographic and clinical characteristics

A total of 331 SLE patients were enrolled in this study, including 300 females (90.63%) and 31 males (9.37%), with a mean age of 35.28 ± 11.37 years and a mean disease duration of 70.79 ± 81.65 months.

### 3.2 Sleep quality characteristics in SLE patients based on LPA

**Figure 1:**
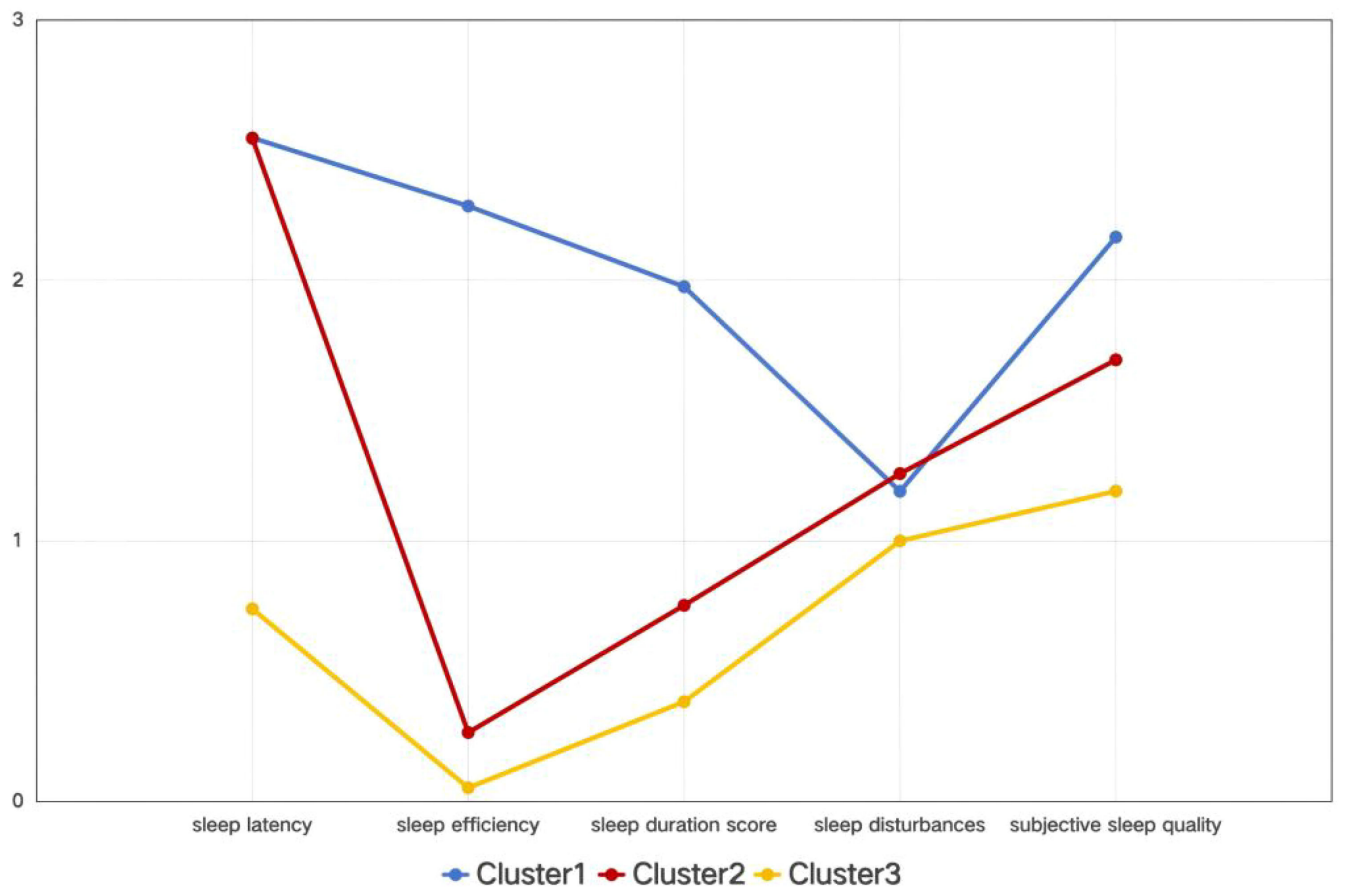
Mean scores of sleep quality domains among different clusters. Based on a cross-sectional latent profile analysis, Cluster 1 was characterized by the highest scores in sleep latency, sleep efficiency, and subjective sleep quality. Cluster 2 exhibited relatively high scores in sleep latency and subjective sleep quality. In contrast, three clusters are presented: Cluster 1 (severe sleep disturbance), Cluster 2 (moderate sleep disturbance), and Cluster 3 (mild sleep disturbance), indicating a gradient difference.

On the basis of the PSQI’s five dimensions, it was established that a three-class latent profile model provided the best fit. LPA yielded an entropy value of 0.996 for the three-cluster model. Entropy is a commonly used measure of classification accuracy, ranging from 0 to 1, with values closer to 1 indicating more precise classification. An entropy value above 0.8 is generally considered acceptable, corresponding to a classification accuracy exceeding 90% (29). The three-cluster model demonstrated the lowest Bayesian information criterion and Akaike information criterion values, indicating the best model fit (30). Cluster 1 was characterized by the highest scores in sleep latency, sleep efficiency, and subjective sleep quality. Cluster 2 exhibited relatively high scores in sleep latency and subjective sleep quality. In contrast, three clusters were presented: Cluster 1 (severe sleep disturbance), Cluster 2 (moderate sleep disturbance), and Cluster 3 (mild sleep disturbance), shoeing a gradient of differences (**Table 1**, **Figure 1**).

**Table 1:** Fit statistics for latent profile analysis models with 1–6 profile solutions.

Class	LogLik	AIC	BIC	aBIC	Entropy	BLRT
1	−1934.00	3888.00	3926.02	3894.29	1.000	-
2	−1832.17	3696.34	3757.18	3706.42	0.875	0.001
3	−1691.08	3131.74	3261.01	3440.01	0.996	0.010
4	−1647.14	3350.28	3456.74	3367.92	0.886	0.010
5	−1531.87	3426.15	3509.80	3153.16	0.899	0.010
6	−1517.35	3114.69	3266.78	3139.89	0.878	0.010

### 3.3 Sleep quality of enrolled sample

The mean total PSQI score for the SLE sample was 7.59 ± 3.44, and the mean values for the individual PSQI components were as follows: component 1 (subjective sleep quality), 1.58 ± 0.76; component 2 (sleep latency), 1.92 ± 1.03; component 3 (sleep duration), 0.78 ± 0.91; component 4 (sleep efficiency), 0.45 ± 0.80; component 5 (sleep disturbances), 1.16 ± 0.51; component 6 (use of sleep medication), 0.24 ± 0.76; and component 7 (daytime dysfunction), 1.48 ± 1.19.The P values for all dimensions and total scores were < 0.05, indicating statistically significant intergroup differences, with the most pronounced differences observed in PSQI–sleep latency and PSQI–sleep efficiency (%) (**Table 2**).

**Table 2:** Average score of each cluster in key areas.

Variable	Overall	Class1(n=42)	Class2(n=174)	Class3(n=115)	Statistic	*P*
PSQI–subjective sleep quality	1.58 ± 0.76	2.17 ± 0.73	1.70 ± 0.68^a^	1.19 ± 0.69^ab^	*36.25*	<0.001
PSQI–sleep latency	1.92 ± 1.03	2.55 ± 0.86	2.55 ± 0.53	0.74 ± 0.44^ab^	*395.18*	<0.001
PSQI–sleep duration	0.78 ± 0.91	1.98 ± 1.07	0.75 ± 0.78^a^	0.38 ± 0.63^ab^	*65.42*	<0.001
PSQI–sleep efficiency (%)	0.45 ± 0.80	2.29 ± 0.46	0.26 ± 0.44^a^	0.05 ± 0.22^ab^	*565.09*	<0.001
PSQI–sleep disturbances	1.16 ± 0.51	1.19 ± 0.51	1.26 ± 0.48	1.00 ± 0.51^ab^	*9.60*	<0.001
PSQI–use of sleep medication	0.24 ± 0.76	0.29 ± 0.89	0.37 ± 0.90	0.03 ± 0.29^ab^	*7.27*	<0.001
PSQI–daytime dysfunction	1.48 ± 1.19	1.71 ± 1.22	1.59 ± 1.19	1.23 ± 1.16^ab^	*4.19*	0.016
PSQI–score	7.59 ± 3.44	12.17 ± 3.34	8.45 ± 2.26^a^	4.61 ± 2.17^ab^	*177.02*	<0.001

a means statistically significant compared with Cluster 1; b means statistically significant compared with Cluster 2.

### 3.4 Descriptive and differential analyses of baseline demographic among clusters

This study enrolled 331 patients who were classified into three clusters based on their characteristics: Cluster 1 (n = 42), Cluster 2 (n = 174), and Cluster 3 (n = 115). Significant differences were observed in the median disease duration among the clusters, with Cluster 2 showing the longest progression (60 months), followed by Cluster 3 (36 months), while Cluster 1 had the shortest duration (17 months). Notably, all divorced patients (two cases) were in Cluster 1. However, no significant differences were found among clusters regarding other variables such as age, gender, BMI, smoking/alcohol history, place of residence, and household income (**Table 3**).

**Table 3:** Descriptive and differential analyses of baseline demographic and disease characteristics among three clusters.

Variable	Total	Cluster1(n=42)	Cluster2(n=174)	Cluster3(n=115)	Statistic	*P*
Age	33.00 [27.00, 41.00]	36.00 [30.00, 49.00]	34.00 [27.00, 43.00]	33.00 [26.00, 40.00]	*H* = 4.91	0.086
Body mass index	21.30 [19.43, 23.05]	22.05 [19.43, 23.56]	21.09 [19.53, 23.24]	21.12 [19.23, 22.86]	*H* = 2.44	0.296
Gender					*χ2 = 0.867*	0.648
Male	31 (9.37)	3 (7.14)	15 (8.62)	13 (11.30)		
Female	300 (90.63)	39 (92.86)	159 (91.38)	102 (88.70)		
Education level					*χ* ^2^ = 14.92	0.005*
Bachelor’s degree or above	69 (20.85)	11 (15.94)	47 (68.12)	11 (15.94)		
High school/technical secondary school	144 (43.50)	14 (9.72)	71 (49.31)	59 (40.97)		
Junior high school or below	118 (35.65)	17 (14.41)	56 (47.46)	45 (38.14)		
Residential location					-	0.692
Country	23 (6.95)	4 (17.39)	14 (60.87)	5 (21.74)		
City	289 (87.31)	35 (12.11)	151 (52.25)	103 (35.64)		
Town	19 (5.74)	3 (15.79)	9 (47.37)	7 (36.84)		
Smoking history					-	0.732
Never	324 (97.89)	41 (12.65)	169 (52.16)	114 (35.19)		
Quit	3 (0.91)	1 (33.33)	2 (66.67)	0 (0.00)		
Frequent	1 (0.30)	0 (0.00)	1 (100.00)	0 (0.00)		
Occasional	3 (0.91)	0 (0.00)	2 (66.67)	1 (33.33)		
Drinking history					-	0.825
Never	317 (95.77)	40 (12.62)	166 (52.37)	111 (35.02)		
Quit	4 (1.21)	0 (0.00)	3 (75.00)	1 (25.00)		
Occasional	10 (3.02)	2 (20.00)	5 (50.00)	3 (30.00)		
Marital status					-	< 0.001*
Divorced	3 (0.91)	2 (66.67)	1 (33.33)	0 (0.00)		
Widowed	1 (0.30)	0 (0.00)	1 (100.00)	0 (0.00)		
Single	251 (72.81)	26 (10.79)	122 (48.96)	103 (40.25)		
Married	76 (22.96)	14 (18.42)	50 (65.79)	12 (15.79)		
Engaged in paid work (within previous 3 months)					-	0.030*
No	4 (1.21)	0 (0.00)	4 (100.00)	0 (0.00)		
Part-time	3 (0.91)	0 (0.00)	3 (100.00)	0 (0.00)		
Other	291 (87.92)	38 (13.06)	143 (49.14)	110 (37.80)		
Full-time	33 (9.97)	4 (12.12)	24 (72.73)	5 (15.15)		
Household monthly income (CNY)					*χ* ^2^ = 1.95	0.924
< 1000	15 (4.53)	3 (20.00)	8 (53.33)	4 (26.67)		
1000–3999	127 (38.37)	16 (12.60)	69 (54.33)	42 (33.07)		
4000–7999	109 (32.93)	15 (13.76)	55 (50.46)	39 (35.78)		
> 8000	80 (24.17)	8 (10.00)	42 (52.50)	30 (37.50)		
Annual medical expenses for SLE (CNY)					*χ* ^2^ = 10.99	0.089
< 1000	17 (5.14)	5 (29.41)	9 (52.94)	3 (17.65)		
1000–3999	47 (14.20)	5 (10.64)	25 (53.19)	17 (36.17)		
4000–7999	34 (10.27)	2 (5.88)	14 (41.18)	18 (52.94)		
> 8000	233 (70.39)	30 (12.88)	126 (54.08)	77 (33.05)		
Disease duration (months)	39.00 [8.00,112.00]	17.00 [3.00, 96.00]	60.00 [10.00, 120.00]	36.00 [7.00, 108.00]	*H* = 6.11	0.047*

### 3.5 Descriptive and differential analyses of disease characteristics, medication use, and laboratory test results among clusters

Significant differences were observed in calcium supplement usage rates among the three groups. Cluster 1 demonstrated the highest calcium supplement utilization rate, while Cluster 2 showed a substantially higher proportion of patients with renal dysfunction compared to the other two groups. Of the 45 patients with renal dysfunction, 30 cases were concentrated in Cluster 2. Regarding psychological symptoms, anxiety and depression incidence rates varied markedly across groups: Cluster 1 had the highest rates whereas Cluster 3 exhibited the lowest. Notably, the rates in Cluster 3 were significantly lower than those in both Cluster 1 and Cluster 2. Cluster 2 also reported the most cases of fatigue cases, while no other differences reached statistical significance (**Table 4**).

**Table 4:** Descriptive and differential analyses of disease characteristics, medication use, and laboratory test results among the three clusters.

Variable	n(%)	Cluster1(n=42)	Cluster2(n=174)	Cluster3(n=115)	Statistic	P
Hormones					*χ* ^2^ = 4.66	0.097
Low dose	49 (14.80)	7 (14.29)	19 (38.78)	23 (46.94)		
Moderate-to-high dose	282 (85.20)	35 (12.41)	155 (54.96)	92 (32.62)		
Immunosuppressants					*χ* ^2^ = 2.78	0.249
No	17 (5.14)	0 (0.00)	11 (64.71)	6 (35.29)		
Yes	314 (94.86)	42 (13.38)	163 (51.91)	109 (34.71)		
Biologics					*χ* ^2^ = 1.43	0.489
No	313 (94.56)	39 (12.46)	167 (53.35)	107 (34.19)		
Yes	18 (5.44)	3 (16.67)	7 (38.89)	8 (44.44)		
Calcium supplements					*χ* ^2^ = 6.02	0.049*
No	90 (27.19)	5 (5.56)	49 (54.44)	36 (40.00)		
Yes	241 (72.81)	37 (15.35)	125 (51.87)	79 (32.78)		
Antihypertensive drugs					*χ* ^2^ = 5.46	0.065
No	263 (79.46)	37 (14.07)	130 (49.43)	96 (36.50)		
Yes	68 (20.54)	5 (7.35)	44 (64.71)	19 (27.94)		
Nonsteroidal anti-inflammatory drugs					-	0.421
No	326 (98.49)	42 (12.88)	172 (52.76)	112 (34.36)		
Yes	5 (1.51)	0 (0.00)	2 (40.00)	3 (60.00)		
Complete blood count					*χ* ^2^ = 5.73	0.057
Abnormal	88 (26.59)	15 (17.05)	51 (57.95)	22 (25.00)		
Normal	243 (73.41)	27 (11.11)	123 (50.62)	93 (38.27)		
Kidney function					*χ* ^2^ = 11.03	0.004*
Abnormal	45 (13.60)	9 (20.00)	30 (66.67)	6 (13.33)		
Normal	286 (86.40)	33 (11.54)	144 (50.35)	109 (38.11)		
Immunoglobulins					*χ* ^2^ = 5.32	0.070
Abnormal	152 (45.92)	25 (16.45)	71 (46.71)	56 (36.84)		
Normal	179 (54.08)	17 (9.50)	103 (57.54)	59 (32.96)		
Complement components 3 and 4					*χ* ^2^ = 1.17	0.558
Abnormal	231 (69.79)	31 (13.42)	117 (50.65)	83 (35.93)		
Normal	100 (30.21)	11 (11.00)	57 (57.00)	32 (32.00)		
24-hour urine protein quantification					*χ* ^2^ = 0.45	0.800
Abnormal	133 (40.18)	16 (12.03)	68 (51.13)	49 (36.84)		
Normal	198 (59.82)	26 (13.13)	106 (53.54)	66 (33.33)		
Urinalysis					*χ* ^2^ = 1.61	0.446
Abnormal	109 (32.93)	17 (15.60)	53 (48.62)	39 (35.78)		
Normal	222 (67.07)	25 (11.26)	121 (54.50)	76 (34.23)		
HADS-anxiety					*χ* ^2^ = 16.45	< 0.001*
No	304 (91.84)	33 (10.86)	158 (51.97)	113 (37.17)		
Yes	27 (8.16)	9 (33.33)	16 (59.26)	2 (7.41)		
HADS-depression					*χ* ^2^ = 9.83	0.007*
No	300 (90.63)	34 (11.33)	155 (51.67)	111 (37.00)		
Yes	31 (9.37)	8 (25.81)	19 (61.29)	4 (12.90)		
Fatigue severity scale					*χ* ^2^ = 13.73	0.001*
No	225 (67.98)	22 (9.78)	111 (49.33)	92 (40.89)		
Yes	106 (32.02)	20 (18.87)	63 (59.43)	23 (21.70)		
SLEDAI-2000 (disease activity)					*χ* ^2^ = 3.96	0.683
No activity	188 (56.80)	24 (12.77)	97 (51.60)	67 (35.64)		
Mild activity	29 (8.76)	3 (10.34)	17 (58.62)	9 (31.03)		
Moderate activity	78 (23.56)	9 (11.54)	38 (48.72)	31 (39.74)		
Severe activity	36 (10.88)	6 (16.67)	22 (61.11)	8 (22.22)		

-, Fisher’s exact test. * The differences between the clusters were statistically significant.

### 3.6 Analysis of factors influencing clusters in latent profile analysis

An unordered multinomial logistic regression model was constructed, with the LPA-derived clusters as the dependent variable and significant factors as independent variables, using Cluster 2 as the reference group. The analysis revealed that disease duration, fatigue severity, calcium supplement use, impaired renal function, anxiety, and depression were all significant factors influencing cluster membership. Compared with Cluster 2, Cluster 3 exhibited lower levels of fatigue [OR (95% CI) = 0.522 (0.437, 0.625)], a lower likelihood of calcium consumption [OR (95% CI) = 0.889 (0.748, 1.056)], a higher likelihood of being free from anxiety [OR (95% CI) = 0.380 (0.231, 0.625)], a smaller family size [OR (95% CI) = 0.938 (0.900, 0.978)], and a longer disease duration [OR (95% CI) = 1.003 (1.002, 1.004)]. In comparison with Cluster 2, Cluster 1 was primarily characterized by a shorter disease course [OR (95% CI) = 0.992 (0.990, 0.994)] and a higher proportion of individuals with undergraduate degrees or above [OR (95% CI) = 2.632 (2.062, 3.359)] (**Table 5**).

**Table 5:** Analysis of factors influencing clusters in latent profile analysis.

Variable	Cluster3(Ref=Cluster2)		Cluster1(Ref=Cluster2)	
	*OR(95CI)*	*P*	*OR(95CI)*	*P*
Intercept	—	0.964	—	0.975
Fatigue severity scale				
Yes	ref		ref	
No	0.522 (0.437, 0.625)	0.000	0.690 (0.542, 0.878)	0.003
Calcium				
Yes	ref		ref	
No	0.889 (0.748, 1.056)	0.180	0.400 (0.290, 0.551)	<0.001
Liver and kidney functions				
Normal	ref		ref	
Abnormal	2.468 (1.823, 3.342)	0.000	1.501 (1.113, 2.024)	0.008
HADS-anxiety				
Yes	ref		ref	
No	0.380 (0.231, 0.625)	0.000	0.380 (0.231, 0.625)	<0.001
HADS-depression				
Yes	ref		ref	
No	0.512 (0.350, 0.748)	0.001	0.759 (0.518, 1.111)	0.157
Baseline (education level)				
High school/technical secondary school	ref		ref	
Bachelor’s degree or above	1.170 (0.858, 1.594)	0.998	2.632 (2.062, 3.359)	<0.001
Junior high school or below	1.536 (1.178, 2.002)	0.902	0.833 (0.703, 0.989)	0.037
Household size (persons)	0.938 (0.900, 0.978)	0.002	1.017 (0.947, 1.092)	0.649
Disease duration	1.003 (1.002, 1.004)	0.000	0.992 (0.990, 0.994)	<0.001

## 4 Discussion

SLE is a common autoimmune disease frequently associated with poor sleep quality. Previous studies have reported that the prevalence of poor sleep quality among SLE patients ranges from 42% to 81% (31–33). Sleep is essential for both physical and mental health, and its impairment can significantly exacerbate disease burden, negatively affecting overall health and quality of life. While good sleep quality can help alleviate fatigue in SLE patients, chronic sleep disturbances may weaken immune function, complicate disease management, and contribute to additional health issues (31, 34). Chronic short sleep duration has been associated with a higher SLE risk, with stronger effects observed among those experiencing bodily pain and depression, highlighting the potential role of adequate sleep in disease prevention (35). A large-scale cohort study indicates that patients with sleep disorders are at a higher risk of developing autoimmune diseases (36).

Research has shown that all domains of sleep quality are affected in SLE patients (37–39). A meta-analysis found that subjective sleep quality and habitual sleep efficiency were the most impaired domains compared to healthy individuals (40). In this study, stratified cluster analysis based on PSQI scores identified three distinct patterns of sleep disturbances in SLE patients: Cluster 1 (severe sleep disturbance), Cluster 2 (moderate sleep disturbance), and Cluster 3 (mild sleep disturbance). Sleep latency and subjective sleep quality were the most affected domains. Self-reported sleep quality may be influenced by various factors, including sociocultural differences, cognitive and memory impairments, and mood disorders (41).

Using Cluster 2 as the reference group, Cluster 3 (mild sleep disturbance) was associated with less severe anxiety symptoms. The impact of negative emotions on sleep disturbances in SLE patients has been well-documented (42–44). Studies indicate that depressive symptoms (45, 46) and other negative emotions (42) are closely linked to the severity of poor sleep quality. Patients with poor sleep quality tended to have higher levels of depression than good sleepers (47). Since the relationship between anxiety symptoms and sleep quality in SLE patients was more clearly demonstrated in this study, the development of strategies to improve sleep quality in patients with SLE underscores the continued importance of mental health assessments.

Compared to Cluster 2, Cluster 3 also had lower fatigue levels. The bidirectional relationship between fatigue and poor sleep quality is well established—sleep deprivation and poor sleep quality increase fatigue, which in turn disrupts sleep patterns. Fatigue is a major contributor to sleep disturbances in SLE patients (48).

Cluster 1 (severe sleep disturbance) scored highest across all seven components, indicating that their sleep problems were comprehensive. The most critical distinguishing feature of Cluster 1 was extremely poor sleep efficiency (a sleep maintenance disorder), which was characterized by difficulty maintaining continuous sleep and frequent nighttime awakenings.

Sleep latency emerged as the most significantly affected domain across all three clusters. Sleep hygiene education plays a critical role in managing sleep disturbances and emphasizes lifestyle modifications, including reducing caffeine, nicotine, and alcohol intake, maintaining regular physical activity, and optimizing the sleep environment (e.g., light, noise, and temperature control). Establishing a consistent sleep schedule is also recommended (49–53).

The impact of disease activity on sleep quality remains controversial (38, 54). Our study found that SLE-related factors such as disease activity, cumulative organ damage, organ involvement, and treatment exposure did not significantly influence sleep patterns.

This analysis shows that personalized treatment approaches are essential for the comprehensive management of SLE, and addressing sleep disturbances should be a key component of this strategy. For Cluster 2, the treatment focus should be on improving difficulties in sleep initiation. For Cluster 1, in addition to addressing sleep initiation problems, it is necessary to intervene in sleep maintenance disorder, as well as the assessment and treatment of other comorbidities. From a precision medicine perspective, identifying distinct sleep disturbance patterns in SLE patients, thereby guiding the feasibility of individualized clinical management (10).

### 4.1 Limitations

The study had several limitations. First, it cross-sectional design, precludes causal inference. Causation should be verified through longitudinal studies. Second the small number of patients using sleep medications prevented a detailed analysis of medication use across clusters. However, all the patients in study were enrolled from our lupus cohort, ensuring comprehensive clinical and treatment data collection. The availability of detailed historical clinical data is a major strength of this research. Despite these limitations, the large sample size provides robust evidence supporting the personalized management of sleep disturbances in SLE patients.

## 5 Conclusions

Future research should aim to validate these findings in larger, multicenter studies with longitudinal designs to better understand the causality and dynamics of sleep disturbances in SLE patients. Furthermore, studies examining the effectiveness of sleep hygiene education and personalized treatment approaches to improve sleep outcomes in SLE patients are warranted. By addressing sleep disturbances, we can potentially enhance the overall quality of life, disease management, and long-term health outcomes for SLE patients.

## Data Availability

The original contributions presented in the study are included in the article/supplementary material. Further inquiries can be directed to the corresponding author.
